# Post-Transcriptional Regulation of Anti-Apoptotic BCL2 Family Members

**DOI:** 10.3390/ijms19010308

**Published:** 2018-01-20

**Authors:** Jia Cui, William J. Placzek

**Affiliations:** Department of Biochemistry and Molecular Genetics, University of Alabama at Birmingham, Birmingham, AL 35294, USA; jiacui@uab.edu

**Keywords:** anti-apoptotic BCL2 subfamily, apoptosis, post-transcriptional regulation, RNA binding proteins, microRNA, alternative splicing, mRNA stability, mRNA subcellular localization

## Abstract

Anti-apoptotic B cell lymphoma 2 (BCL2) family members (BCL2, MCL1, BCLxL, BCLW, and BFL1) are key players in the regulation of intrinsic apoptosis. Dysregulation of these proteins not only impairs normal development, but also contributes to tumor progression and resistance to various anti-cancer therapies. Therefore, cells maintain strict control over the expression of anti-apoptotic BCL2 family members using multiple mechanisms. Over the past two decades, the importance of post-transcriptional regulation of mRNA in controlling gene expression and its impact on normal homeostasis and disease have begun to be appreciated. In this review, we discuss the RNA binding proteins (RBPs) and microRNAs (miRNAs) that mediate post-transcriptional regulation of the anti-apoptotic BCL2 family members. We describe their roles and impact on alternative splicing, mRNA turnover, and mRNA subcellular localization. We also point out the importance of future studies in characterizing the crosstalk between RBPs and miRNAs in regulating anti-apoptotic BCL2 family member expression and ultimately apoptosis.

## 1. Introduction

Apoptosis is a physiological process of programmed cell death that maintains tissue function and homeostasis. It is generally characterized by several morphological features including cell shrinkage, chromatin condensation, membrane blebbing, and the formation of apoptotic bodies under light and electron microscopy [1]. Tightly regulated apoptosis plays important roles in multiple aspects of both normal physiology, such as development [2] and immune responses [3,4], and disease biology such as neurodegenerative diseases [5,6], cardiovascular diseases [7,8], carcinogenesis [9,10], and treatment responses to certain drugs [9,11].

Apoptosis is tightly regulated by two major pathways: the extrinsic/death receptor pathway and the intrinsic/mitochondrial pathway. The extrinsic apoptosis pathway is triggered by ligand–receptor interactions between tumor necrosis factor (TNF) family members and corresponding transmembrane death receptors. Once ligands bind, adaptor proteins are recruited and bind to death receptors, resulting in activation of downstream caspase cascades and ultimately cell death. For more details, see [12].

**Figure 1:**
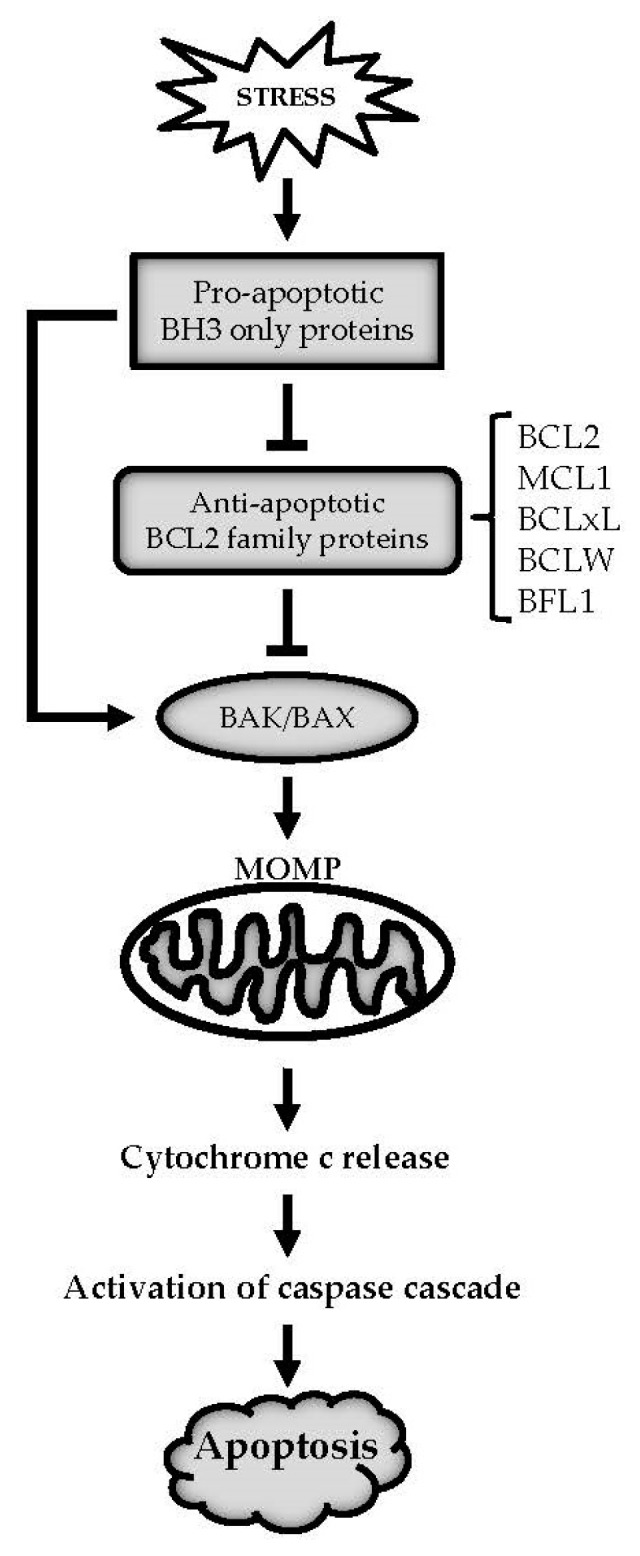
B cell lymphoma 2 (BCL2) family mediated intrinsic apoptosis signaling. Under cellular stress, pro-apoptotic BCL2 homology 3 (BH3) only proteins are activated, which directly activates BAX/BAX and/or neutralizes the anti-apoptotic BCL2 family members such as BCL2, MCL1, BCLxL, BCLW, and BFL1. This in turn releases BAK/BAX to oligomerize on the mitochondria and results in mitochondrial outer membrane permeabilization (MOMP). The resulting MOMP causes cytochrome c release, which then activates downstream caspase cascade and ultimately triggers apoptosis.

The intrinsic apoptosis pathway is mediated by the B cell lymphoma 2 (BCL2) family of proteins [13]. The BCL2 family, whose namesake was initially identified due to chromosomal translocations that activated *BCL2* gene expression [14], has been identified through their composition of a series of shared BCL2 homology (BH) motifs (BH1, BH2, BH3, and BH4). Importantly, only the BH3 motif, which mediates protein–protein interactions between the family members, is strictly conserved across all family members. These proteins are divided into three major subfamilies based on their function and structure: the anti-apoptotic BCL2 proteins (BCL2, MCL1: myeloid cell leukemia 1; BCLxL: B-cell lymphoma extra large; BCLW: BCL2-like protein 2, and BFL1: BCL2-related protein A1), the pro-apoptotic effectors (BAK: BCL2 homologous antagonist/killer; BAX: BCL2-associated X; and BOK: BCL2-related ovarian killer), and the pro-apoptotic BH3 only proteins [15]. The BH3 only proteins can be subclassified into direct activators and sensitizers based on their binding ability to other BCL2 family members [13]. The direct activator BH3 only proteins (e.g., BID: BH3 interacting-domain death agonist and BIM: BCL2-like protein 11) can interact with both the anti-apoptotic BCL2 proteins and the effectors BAK/BAX while the sensitizer BH3 only proteins (e.g., BAD: BCL2-associated death promoter; NOXA: Phorbol-12-myristate-13-acetate-induced protein; and PUMA: p53 upregulated modulator of apoptosis) preferentially bind to the anti-apoptotic BCL2 proteins and thereby indirectly activate BAK/BAX [13]. Cell fate is determined by the balance between the activities of pro-apoptotic and anti-apoptotic BCL2 family members. Under normal cellular homeostasis, the anti-apoptotic members directly bind to the effectors BAK/BAX to counteract their ability to induce apoptosis. However, following cellular stress, such as that induced by radiation or treatment with cytotoxic agents, the BH3 only members are activated. These in turn directly activate BAK/BAX and/or neutralize the function of the anti-apoptotic BCL2 family members by competing for their binding with BAK/BAX. As a result, the effectors BAK/BAX oligomerize in the mitochondrial outer membrane and form pores. The resulting mitochondrial outer membrane permeabilization (MOMP) results in cytochrome c release, activation of downstream caspases, and finally cell death. In addition to their regulation of mitochondrial apoptosis, mediated by the crosstalk among BCL2 family members, BCL2 family proteins have also been shown to induce apoptosis via their regulation of Ca^2+^ signaling [16,17]. A simplified intrinsic apoptosis pathway is highlighted in Figure 1. For more details see [13,15].

The anti-apoptotic BCL2 family of proteins is commonly dysregulated in human cancers through multiple mechanisms including genomic amplification and genetic overexpression [18]. During tumorigenesis and cancer progression, cancer cells rely on upregulation of the anti-apoptotic BCL2 family of proteins and tend to be addicted to these survival mechanisms [19,20]. For example, a t(14;18) chromosomal translocation leads to the amplification of the *BCL2* gene in follicular lymphoma [14]. Similarly, somatic copy number amplification of both the *MCL1* and *BCLxL* genes have been detected across human cancers [21]. Transcriptional activation of the BCL2 family is controlled by multiple proteins. For instance, MCL1 transcription is activated by various transcription factors such as signal transducer and activator of transcription 3 (STAT3), STAT5, hypoxia-inducible factor 1 (HIF1), and E2F1 in a large variety of cancer types [19]. Further, recent studies have begun to identify feedback of the BCL2 family on transcriptional factors that induce their expression [22]. Due to their importance in normal physiology and disease biology especially cancers, regulatory mechanisms of the anti-apoptotic BCL2 family members are being widely studied with several reviews focused on their transcriptional controls [18,19] and post-translational modifications [23,24,25].

Improved genomic technologies and understanding of the importance of non-coding RNA on gene expression have highlighted the multiple mechanisms utilized by cells to regulate gene expression between transcription of the mRNA product and translational protein production. This post-transcriptional regulation of gene expression includes precursor mRNA (pre-mRNA) editing and maturation such as 5′ capping, 3′ polyadenylation, and alternative splicing; mRNA stability control; and mRNA transportation and localization control [26]. It is a dynamic and complicated system mediated by a number of RNA binding proteins (RBPs) and non-coding RNAs such as microRNAs (miRNAs). Understanding the post-transcriptional control of the BCL2 family can complete a comprehensive view of how BCL2 family members are regulated at different levels during gene expression. Further it will help us better understand how such dysregulated post-transcriptional control leads to disease biology, especially tumorigenesis, and make use of this knowledge to better target the anti-apoptotic BCL2 family members in cancer treatment. In this review, we will discuss the RBPs and miRNAs that mediate post-transcriptional regulation of the anti-apoptotic BCL2 family members and their corresponding identified roles in normal physiology and diseases.

## 2. RNA Binding Proteins (RBPs) and Anti-Apoptotic BCL2 Family Members

RBPs are a group of proteins containing multiple RNA binding modules that bind to single or double stranded RNAs [27]. By far the most common RNA binding module is the RNA recognition motif (RRM) [27]. The K-homology (KH) domain is also a conserved RNA binding module found in a number of RBPs [27]. The presence of multiple RNA binding modules together with the diversity of RNA binding sites on target RNA create an intricate system for controlling gene expression at the post-transcriptional level [27]. In this section, we will review the RBP-mediated post-transcriptional regulation of the anti-apoptotic BCL2 members with a focus on their identified roles in the control of mRNA splicing, stability, and subcellular localization controls.

### 2.1. Regulation of Pre-mRNA Alternative Splicing

**Figure 2:**
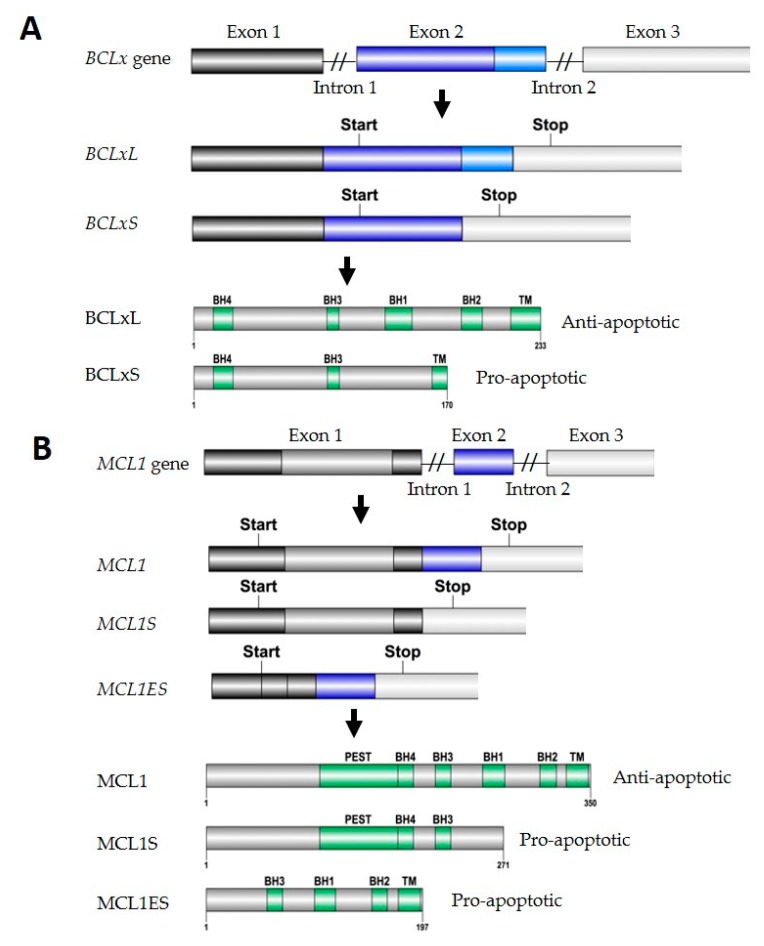
Alternative splicing of *BCLx* and *MCL1*. Each panel represents an alternative splicing process from a pre-mRNA containing both introns and exons, to alternatively spliced mature mRNA isoforms, to different multi-motif protein products (L, long; S, short; ES, extra short). The 3′ end of each gene is truncated. The translational start and stop sites are specified on each alternatively spliced mature mRNA variant. Lastly, the impact of each protein product on apoptosis regulation is listed.

Alternative splicing employs a highly ordered collection of RNA-RNA, protein-protein, and protein–RNA interactions to alter exon inclusion in mature mRNAs. This enables production of multiple protein variants from a single pre-mRNA and is an efficient way to increase the complexity of gene expression in cells. Pre-mRNA splicing is catalyzed by the spliceosomal small nuclear ribonuclear proteins (snRNPs) which bind to the 5′ splice site, 3′ splice site, and branch site in a pre-mRNA (reviewed in [28]). Pre-mRNA also contains *cis*-acting elements including exonic and intronic splicing enhancers or silencers associated with multiple regulatory RBP proteins that either enhance or repress spliceosomes assembled at the splice sites (reviewed in [29,30]). Thus far, several spliceosome components and regulatory RBPs have been identified to modulate variant splicing of two BCL2 family members, *BCLx* and *MCL1*. The splicing control of *BCLx* and *MCL1* is central for regular apoptotic response in normal development with aberrant control of these proteins leading to tumorigenesis, cancer progression, and drug insensitivity. The splicing variants and the corresponding protein products of *BCLx* and *MCL1* are summarized in Figure 2.

#### 2.1.1. *BCLx* Splicing

*BCLx* can be alternatively spliced to produce either a long isoform, *BCLxL*, that contains three exons; or a short isoform, *BCLxS*, in which the 3′ portion of exon 2 is spliced out [31]. The mature *BCLxL* and *BCLxS* retain a shared 3′ untranslated region (UTR). The two protein products BCLxL and BCLxS have opposite functions wherein BCLxL is anti-apoptotic and BCLxS, losing the BH1 and BH2 motifs, is pro-apoptotic [31]. In vivo studies have demonstrated that neonatal hypoxia-ischemia induces *BCLx* splicing toward its short form, which increases the expression of the BCLxS variant in the rat hippocampus and cortex [32]. In colorectal cancers, BCLxL has been identified to be significantly upregulated in both adenoma and adenocarcinoma tissues compared with normal mucosa [33]. However, the expression profile of BCLxS has been poorly explored in vivo in cancer or other disease models.

Thus far, the regulatory mechanisms of *BCLx* splicing have been largely investigated using in vitro biochemistry methods or cell lines. Recently, a genome-wide small interfering RNA (siRNA) screen using a *BCLx* splice reporter construct in HELA cells identified several components of the spliceosome complex that are directly involved in *BCLx* splicing. The identified snRNP and splicing factors (SF) include: the U1 snRNP protein (SNRP70); U2AF1; U2 snRNP proteins (SF3B1, SF3B4, SF3B5, SF3A1, SF3A3, SNRNPA1); U5 snRNP proteins (U5-200K, PRPF6, UPS39); spliceosomal (Sm) and like Sm (LSM) core proteins (SmB/B′, SmD1, SmD2, SmD3); and survival motor neuron (SMN) complex proteins (SMN1, GEMIN4: Gem-associated protein 4) [34]. It was further determined, using western blot analysis and RT-qPCR, that knockdown of SF3B1 favors *BCLx* splicing to produce more *BCLxS* [34]. This alternative splicing network plays an important role in cell cycle control under mitotic stress [34]. In addition to the direct splicing factors, other regulatory RBPs have also been identified to enhance or inhibit *BCLx* splicing. We have grouped RBPs according to four families— heterogeneous nuclear ribonucleoprotein (hnRNP) proteins, serine/arginine-rich (SR) proteins, signal transduction and activation of RNA (STAR) proteins, and RNA binding motif (RBM) proteins. These RBPs and their impact on *BCLx* splicing are outlined in Table 1.

##### hnRNP Proteins

Heterogeneous nuclear ribonucleoproteins (hnRNPs) are a large family of RBPs that control multiple processes in RNA metabolism [52]. More than 20 hnRNPs have thus far been identified; each containing one or more RNA binding modules such as RRMs, KH domains, qRRMs (quasi-RNA recognition motifs), etc. [52]. The functions of hnRNPs are largely determined by their RNA binding ability and subcellular localization [52]. Thus far, six hnRNP family members have been characterized regarding their impact on *BCLx* splicing, with four promoting production of *BCLxS* and two *BCLxL*. In vitro studies have shown that hnRNP F and H interacted with a 30-nucleotide G-rich element in *BCLx* and improved the production of the *BCLxS* variant [39]. Consistent with the in vitro data, knockdown of hnRNP F and H using siRNAs in cells decreases the *BCLxS*/*BCLxL* ratio from either the plasmid-derived *BCLx* transcript or an endogenously expressed *BCLx* transcript [39]. Likewise, hnRNP K associates with *BCLx* RNA and enhances production of the *BCLxS* isoform in both PC3 and HELA cells [40]. hnRNP I/PTBP1 (polypyrimidine tract binding protein 1), another hnRNP family protein, directly binds to a polypyrimidine tract between two alternative 5′ splice sites on *BCLx* [41]. Overexpression of PTBP1 promotes distal 5′ splice site selection in *BCLx* exon 2 to generate the *BCLxS* splice variant while depletion of PTBP1 enhances the production of *BCLxL* [41]. Mechanistically, this 5′ splice site selection is due to the competition of PTBP1 with a SR protein SRSF1 binding for *BCLx* RNA [41]. Alternatively, interference with hnRNP A1 nuclear to cytoplasmic shuttling activity decreases the levels of *BCLxL* in normal and BCR/ABL-transformed myeloid progenitor cells [35]. In addition to interactions with other RBPs, hnRNP family member activity is also impacted by interactions with long non-coding RNA (lncRNA). It has been demonstrated that in breast cancers, the lncRNA, BC200, is oncogenic as its knockdown facilitates expression of the pro-apoptotic *BCLxS* isoform [36]. Mechanistically, BC200 binds to *BCLx* via a 17-nucleotide complementary sequence and recruits hnRNP A2B1, thereby impairing the association of *BCLx* with the *BCLxS*-promoting splicing factor SAM68 (Src-associated in mitosis of 68 kDa) (discussed in STAR Proteins section, below) and reducing the production of the *BCLxS* variant [36]. A number of additional hnRNP family members (hnRNP C, hnRNP D, and hnRNP H2) were recently identified to target *BCLx* [34], though more detailed analysis of these hnRNP family members is required to characterize their roles in regulating *BCLx* alternative splicing.

##### SR Proteins

The serine/arginine-rich protein family is a group of RBPs that contain a characteristic C-terminal arginine and serine rich domain (RS domain) and one or two N-terminal RRMs [53]. This protein family has been reported to play essential roles in splicing by promoting or suppressing the function of other splicing factors. Thus far a number of SR proteins have been identified as key regulators of *BCLx* splicing. SRSF10 has been shown to promote *BCLxS* production [42]. Following DNA damage, interactions of SRSF10 with other splicing factors, such as the hnRNP family proteins hnRNP K and hnRNP F/H, change; this alters the alternative splicing of a number of DNA damage response transcripts in apoptosis, cell cycle control, and DNA repair [42]. SC35, another member of the SR protein family, has been reported as a key direct target of the transcription factor E2F1. Following its expression, SC35 switches the cellular splicing profile, including that of *BCLx* towards the pro-apoptotic *BCLxS* splicing variant [43]. Alternatively, SRp30C has been characterized to bind to ML2 (HincII and XL+MscI treated downstream fragment) and AM2 (AccI and MscI treated downstream fragment) elements in *BCLx* exon 2 and shifts the splicing to the *BCLxL* variant by stabilizing U1 snRNP at the 5′ splice site [38]. Pinin (PNN), a serine/arginine related protein, has been shown to alter the expression and function of SR proteins wherein PNN depletion diminished expression of SR proteins [37]. Specifically, PNN loss alters SRSF1-mediated *BCLx* splicing to produce more of the *BCLxS* isoform and induces apoptosis in MCF7 cells [37]. Genome-wide siRNA screening has implicated the SR family proteins SRSF2B and SRSF3 in *BCLx* splicing during early spliceosome formation [34], but more detailed studies are required to characterize their roles in *BCLx*-splicing-mediated apoptosis.

##### STAR Proteins

The signal transduction and activation of RNA (STAR) family is a conserved group of KH-domain-containing RBPs [54]. In addition to the KH domain, STAR proteins contain a STAR domain, which is responsible for protein homodimerization and RNA recognition [55]. Like hnRNP and SR proteins, STAR proteins also regulate different aspects in RNA metabolism and processing including pre-mRNA alternative splicing. STAR proteins have been shown to be modified by multiple signaling pathways including phosphorylation [56], methylation [57], acetylation [58], and SUMOylation [59]. Thus, they are believed to play significant roles in linking cell signal transduction with RNA metabolism. Thus far among all the STAR family members, SAM68 is the only STAR protein with well characterized regulation of *BCLx* splicing. SAM68 was firstly identified to bind to *BCLx* RNA and regulate its alternative splicing and apoptosis function two decades ago [44]. Depletion of SAM68 induces accumulation of the anti-apoptotic *BCLxL* isoform whereas overexpression of SAM68 favors the pro-apoptotic *BCLxS* isoform [44]. Tyrosine phosphorylation (Y528) of SAM68 by FYN (tyrosine-protein kinase Fyn) suppresses SAM68 regulation of BCLx and favors *BCLxL* splice selection [44]. FYN control of *BCLx* splice factors has further been studied in the pancreatic cancer BxPc3 cell line, where it was shown that inhibition of FYN not only reduces SAM68 phosphorylation, it also decreases hnRNP A2B1 expression [60]. As downregulation of hnRNP A2B1 also favors *BCLxS* splicing, this study suggests that hnRNP A2B1 and SAM68 synergistically regulate *BCLx* splicing and apoptosis in pancreatic cancer [60]. Furthermore, SAM68 has been reported to interact with hnRNP A1 and disruption of the SAM68–hnRNP A1 interaction attenuates *BCLxS* splicing [44]. Other factors involved in SAM68-mediated *BCLx* splicing have also been reported. For example, protein arginine N-methyltransferase 2 (PRMT2) interacts with SAM68 and increases the BCLxL/BCLxS ratio in TNF-α or lipopolysaccharide (LPS) stimulated cells, suggesting a role during inflammatory response [61]. Also, the transcription factor FBI-1 (factor binding IST protein 1) interacts with SAM68 and reduces SAM68 binding to *BCLx*, thereby inhibiting SAM68-mediated *BCLx* alternative splicing to produce more anti-apoptotic variant *BCLxL* and suppressing Sam68-induced apoptosis [62]. Other STAR proteins such as T-STAR [63] and ASD-2 (alternative splicing defective protein 2) [64] have also been identified as splicing regulators, but more studies are necessary to pursue their roles in regulating *BCLx* alternative splicing.

##### RBM Proteins

RNA binding motif (RBM) containing proteins are not classified as a protein family but are designated RBM proteins due to the presence of one or more RRMs [47]. Multiple RBM proteins have been identified as novel apoptosis modulators [65]. RBM25 has been shown to specifically interact with a CGGGCA sequence in exon 2 of *BCLx* and promote the pro-apoptotic *BCLxS* 5′ splice site selection [48]. Likewise, RBM11 has been identified as a tissue-specific splicing regulator during neuron and germ cell differentiation [47]. In vitro and in vivo binding assays reveal that RBM11 binds to *BCLx* exon 2 at a sequence downstream of the 5′ distal splicing site through its RRM domain [47]. Splicing assays demonstrate that RBM11 facilitates *BCLx* splicing to produce more *BCLxS* variant and antagonizes the SR protein SRSF1 in modulating *BCLx* splicing [47]. Another RBP protein, RBM10 has been shown to promote a 5′ internal splicing site selection of *BCLx* and RBM10 knockdown reduces the levels of *BCLxS* variant [46]. Recently, RBM4 has been reported as a novel tumor suppressor by controlling cancer-related splicing [45]. Specifically, RBM4 induces cancer cell apoptosis by modulating *BCLx* splicing and shifting preference for the anti-apoptotic *BCLxL* to the pro-apoptotic *BCLxS* [45] isoform. Additionally, RBM4 can also antagonize the oncogenic SR protein SRSF1 to regulate *BCLx* splicing and inhibit cancer cell growth [45].

#### 2.1.2. MCL1 Splicing

MCL1 has been reported to have three splicing variants: *MCL1*, *MCL1S*, and *MCL1ES*. *MCL1* is the predominantly expressed isoform with three exons and its protein product, MCL1, is anti-apoptotic with one N-terminal PEST domain, four BH motifs, and one C-terminal transmembrane (TM) domain. *MCL1S* results from splice removal of exon 2 during mRNA processing to produce a pro-apoptotic protein, MCL1S, that lacks the C terminal BH2 motif and TM domain [66]. It has been shown that MCL1S, unlike MCL1, cannot interact with diverse pro-apoptotic BCL2 family proteins but rather dimerizes with MCL1, and works as a novel pro-apoptotic BH3 only protein in cells [66]. *MCL1ES* is another splicing variant that results from splicing removal of part of exon 1 and encodes a pro-apoptotic protein MCL1ES without the N-terminal PEST domain and BH4 motif [67]. MCL1ES has been shown to localize in cytoplasm with a significant concentration near mitochondria, interact with MCL1 but not BAK and BAX, and be able to induce mitochondrial cell death [67]. However, thus far no splicing factors have been identified that regulate *MCL1ES* production. Although an increase in MCL1 expression plays an important role during cell differentiation [68] and MCL1 is known to be overexpressed in multiple cancer types, the expression profile of the other two splicing variants (MCL1S and MCL1ES) in vivo has been largely ignored. The regulatory mechanisms of *MCL1* splicing thus far have been investigated in vitro using multiple cell lines. A list of direct spliceosome components and regulatory splicing factors that regulate *MCL1* splicing is summarized in Table 1.

*MCL1* splicing to either the anti-apoptotic *MCL1* or pro-apoptotic *MCL1S* isoforms are not as well studied as *BCLx* splicing, but still a number of spliceosome components and regulatory RBPs have been characterized to modulate *MCL1* splicing. A genome-wide siRNA screen using *MCL1* splice reporter constructs in HELA cells identified several spliceosome components (U2 snRNPs–SF3A1, SF3A3, SF3B1, SF3B4, SNRNPA1; Lsm/Sm core–SmB/B′, SmD1, SmD2, SmD3; U5 snRNPs–U5-299K, SRPF6) that are involved in *MCL1* splicing with some of them working coordinately to regulate both *BCLx* and *MCL1* splicing [34]. Recent identification that BCL2 targeted therapeutics, such as ABT-737 [69,70] or ABT-199 [71], could be resistant through upregulation of MCL1, has been exploited to further identify factors that control MCL1 expression. In neuroblastomas, a coupled siRNA screen identified that knockdown of splice factors UBL5 (ubiquitin-like protein 5), PRPF8 (pre-mRNA-processing-splicing factor 8), and SART (squamous cell carcinoma antigen recognized by T-cells) could restore sensitivity to ABT-737 in MCL1-dependent neuroblastomas and increased *MCL1* splicing to produce more *MCL1S* variant [51]. Knockdown or biochemical inhibition of the spliceosome component U2 snRNP SF3B1 also favors *MCL1* splicing toward the pro-apoptotic *MCL1S* variant and sensitizes MCL1-dependent neuroblastoma cells to ABT-737 [51].

Of the above identified RBP families, the SR proteins have thus far been most strongly characterized as having important roles in regulating *MCL1* splicing. Maintaining a similar anti-apoptotic effect on MCL1 as it did to BCLx, knockdown of SRSF1 significantly increases the levels of *MCL1S* isoform in both MCF7 and MDA-MB-231 breast cancer cell lines, and the JAr choriocarcinoma cell line [49]. In clear cell renal cell carcinomas, SRSF2 is commonly decreased and knockdown of SRSF2 in renal cancer-derived cell lines results in decreased expression of *MCL1S* isoforms and inhibiting the apoptotic pathways [50]. Epigenetic modulators have also been characterized to modulate *MCL1* alternative splicing [72]. Nonphosphorylated histone deacetylases 1/2 (HDAC1/2) have been shown to be recruited to pre-mRNA by binding to splicing factors, such as SRSF1, and act with lysine acetyltransferases (KATs), such as KAT2B, to catalyze histone acetylation of histone 3 (H3) and histone 4 (H4) of the histone 3 lysine 4 (H3K4) methylated *MCL1* exon 2 [72]. As a result, HDAC1/2 activation regulates *MCL1* alternative splicing to produce more anti-apoptotic *MCL1* [72]. More research needs to be performed to characterize the roles of other RBPs and their upstream signaling regulators in modulating *MCL1* splicing.

### 2.2. Regulation of mRNA Stability

In addition to splice regulation, mRNA turnover plays a critical role in regulating gene expression and quality control of RNA during biogenesis. Typically, mRNA decay is modulated by the RNA-stabilizing or RNA-destabilizing elements on a RNA and is influenced by RBPs bound to these elements [73]. These *cis*-acting elements are commonly located in the 3′ untranslated region (UTR) of a RNA [73]. The anti-apoptotic BCL2 family members contain 3′ UTRs of variable lengths (1506 to 5278 nt), and maintain significant variation in their mRNA half-lives (Table 2). Thus far, several *cis*-acting elements have been identified in the 3′ UTR of anti-apoptotic BCL2 family members including AU-rich elements, CU-rich elements, and GU-rich elements. All have been shown to regulate mRNA stability in a positive or negative fashion depending on the direct interactions they mediate with RBPs. RBPs that act as *BCL2*, *MCL1*, and *BCLxL* mRNA stabilizers or destabilizers are summarized in Table 2.

#### 2.2.1. AU-Rich-Element (ARE) Mediated RNA Decay

AU-rich elements are the most studied mRNA stabilizing/destabilizing elements. The ARE-binding protein HuR (human antigen R) has been reported to bind to the 3′ UTRs of *MCL1*, *BCL2*, and *BCLxL*, stabilizing their RNA, and increasing their expression in glioma [74]. This post-transcriptional regulation promotes glioma growth and chemoresistance to standard treatments including etoposide, topotecan, and cisplatin [74]. In HELA cells, similar data have identified that HuR binds to *MCL1* and *BCL2* RNA with knockdown of HuR decreasing MCL1 and BCL2 expression, thereby inducing apoptosis [75]. Several in vitro and in vivo assays have identified that nucleolin binds to the ARE elements in both *BCL2* and *BCLxL* 3′ UTRs, thereby stabilizing both *BCL2* and *BCLxL* anti-apoptotic messages [76,77]. This stabilization of *BCL2* and *BCLxL* has been identified to control apoptosis and cell survival in multiple normal and cancer cells [76,77,78,79,80]. Several other ARE-binding proteins have also been identified to stabilize *BCL2*. For example, ζ-crystallin, TRA2β (transformer 2β), and LARP1 (La-related protein 1) have been shown to stabilize *BCL2* and enhance BCL2 expression in T cell acute lymphocytic leukemia, colon cancers, and ovarian cancers, respectively [81,82,83]. Alternatively, several ARE-binding proteins have been characterized to decrease mRNA stability. In pathogen-engaged neutrophils, the RNA-destabilizing protein tristetraprolin (TTP) regulates apoptosis during bacterial infection by binding to the ARE elements at the *MCL1* 3′ UTR, destabilizing *MCL1*, and decreasing MCL1 expression [84]. Additionally, AUF1 (AU-rich element RNA-binding protein 1) and ZFP36L1 (zinc finger protein 36, C3H1 type-like 1) have been shown to destabilize *BCL2* and decrease BCL2 expression [85,86,87]. Further studies characterizing the interplay of these AU-rich binding proteins with one another and identification of the members responsible for regulation of BCLW and BFL1 are needed.

**Table 2:** A summary of *BCL2*, *MCL1*, and *BCLxL* mRNA half-lives in different cell lines and their mRNA stabilizers and destabilizers.

mRNA	Half-Life	Cell Type	mRNA Stabilizer	mRNA Destabilizer
*BCL2*	>6 h [74]	U251	HuR [74], nucleolin [76], ζ-crystallin [81], TRA2β [82], LARP1 [83]	AUF1 [85], ZFP36L1 [87]
	5 h [81]	Jurkat		
	7.5 h [81]	HEK 293		
	8 h [82]	HCT116		
	2.5 h [87]	Murine leukemia BCL1 cells		
*MCL1*	1.4 h [88]	PC3	HuR [74], CUGBP2 [89]	PTBP1 [88], TTP [84]
	2.0 h [88]	H1299		
	4 h [74]	U251		
	2.3 h [84]	Mice peritoneal neutrophil stimulated with LPS		
	0.5 h [89]	HCT116		
*BCLxL*	3.4 h [74]	U251	HuR [74], nucleolin [77]	n.a
	4 h [77]	HELA		
	>6 h [78]	PAEC		

n.a: not available.

#### 2.2.2. CU-Rich-Element Mediated RNA Decay

PTBP1 is a RBP that binds to CU-rich elements within RNA. PTBP1 has been reported to bind to *MCL1* especially at its 3′ UTR and act to destabilize *MCL1* mRNA [88]. Knockdown of PTBP1 has further been shown to increase MCL1 expression in multiple cancer cell lines due to its stabilization of *MCL1* [88]. Further studies identified that the pro-apoptotic effect of PTBP1 under mitotic stress induced by antitubulin drugs vincristine and docetaxel results from its suppression of MCL1 expression in cancer cells [88]. Thus far no other RBPs binding to CU-rich elements have been reported to regulate the half-lives of either *MCL1* or other anti-apoptotic BCL2 family members.

#### 2.2.3. GU-Rich-Element (GRE) Mediated RNA Decay

CUGBP2 (CUG triplet repeat RNA-binding protein 2) is a GRE binding protein. Studies have shown that MCL1 is a novel target of CUGBP2 wherein CUGBP2 binds to the *MCL1* 3′ UTR and increases *MCL1* mRNA stability in colon cancers [89]. However, overexpression of CUGBP2 in colon cancer cells reduces MCL1 protein levels possibly due to its inhibitory effect on translation [89].

### 2.3. Regulation of mRNA Subcellular Localization

mRNA localization is a conserved mechanism that employs proteins to localize mRNA transcripts and proteins to their site of function [90]. It is regulated by the dynamic remodeling of ribonucleoprotein (RNP) complexes via interactions between different *trans*-acting proteins and *cis*-acting mRNA sequences [90]. mRNA localization plays important roles in effective and precise protein translation and further regulates diverse cellular functions, including neuronal morphogenesis [91]. Characterization of the proteins that regulate BCL2 family mRNA has begun to identify some of the regulators of their subcellular localization. The first study to characterize RBP impact on BCL2 family mRNA localization demonstrated that HuR silencing in glioma cells results in a general shift of *MCL1*, *BCL2*, and *BCLxL* mRNA towards polyribosomes [74]. This indicates that HuR not only regulates the degradation of *MCL1*, *BCL2*, and *BCLxL* mRNA, but it also controls their translation [74]. Similarly, it has been shown that knockdown of PTBP1 increases *MCL1* mRNA accumulation in the cytoplasm in prostate and lung cancer cells [88]. SFPQ (splicing factor, proline- and glutamine-rich) has been shown to bind to *BCLW* mRNA at its 3′ UTR and it is required for axonal trafficking of *BCLW* [92]. This SRPQ-mediated spatial gene expression is essential to promote axonal viability [92]. It is likely that a number of the RBPs identified in the previous two sections also impact subcellular localization of mRNA. However, thus far characterization of this portion of their effects has been largely ignored.

## 3. MicroRNAs (miRNAs) and Anti-Apoptotic BCL2 Family Members

**Figure 3:**
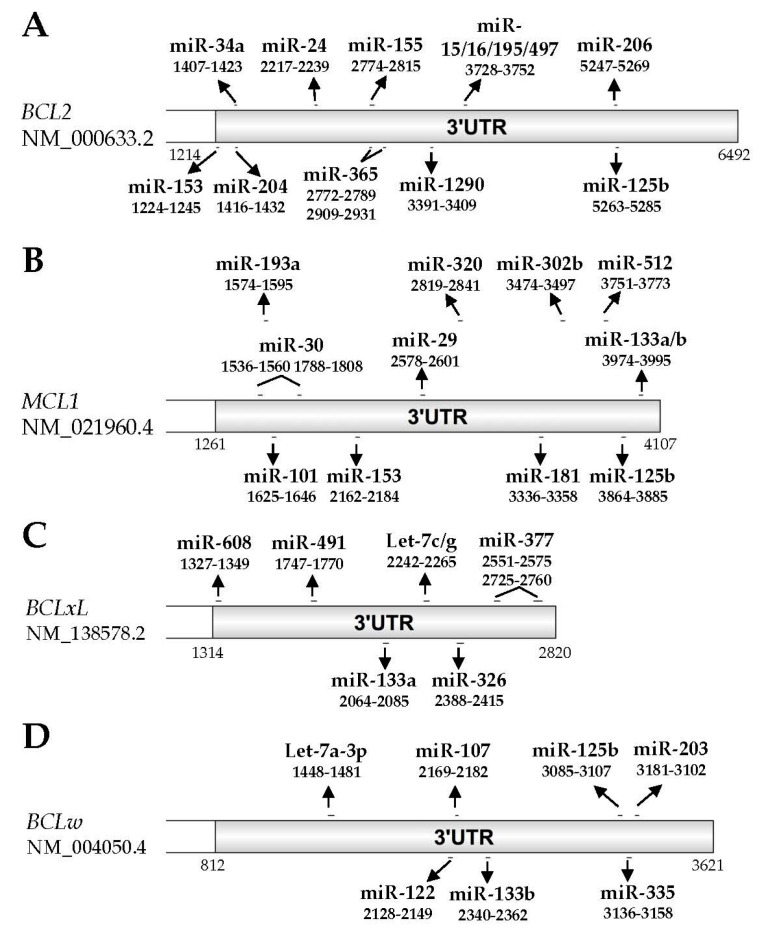
Validated miRNA binding sites on the 3′ untranslated region (UTR) of anti-apoptotic family members. A schematic diagram of 3′ UTR of *BCL2* (NM_000633.2, nucleotides 1214–6492), *MCL1* (NM_021960.4, nucleotides 1261–4107), *BCLxL* (NM_138578.2, nucleotides 1314–2820), and *BCLW* (NM_004050.4, nucleotides 812–3621). Validated miRNAs and their binding positions to each mRNA transcript are shown in line.

In addition to RBPs, miRNAs are another group of molecules in cells that control gene expression post-transcriptionally. A number of studies have connected RBPs with miRNAs in regulating gene expression with RBPs either enhancing or counteracting miRNA targeting of a shared mRNA [93], though limited analysis of RBP impact on miRNA targeting of the BCL2 family has been conducted. miRNAs are small non-coding RNAs of around 22 nucleotides in length and are highly conserved in various organisms. miRNAs function via perfect or imperfect base-pairing with their target mRNAs typically in the 3′ UTR. Mechanistically, mature miRNAs are loaded onto the miRNA induced silencing complex (miRISC) where the core component argonaute (AGO) family proteins recruit other factors to induce target mRNA degradation or translational repression. For more details about the molecular mechanisms of miRNA-mediated gene silencing, see ref [94]. Over the past two decades, miRNAs have been identified as key regulators in cancer biology. Numerous studies have demonstrated that miRNA-mediated dysregulation of gene expression is involved in multiple aspects in cancer biology such as tumor cell growth, cell death resistance, dysregulated metabolism, invasion, metastasis, immune evasion, and so on (reviewed in [95]). Thus far, more than 35 miRNAs have been identified to target and decrease the expression of the anti-apoptotic BCL2 family members (Table 3 and Figure 3). All miRNAs referenced in this review were validated in the cited literature by assessing their impact on both the endogenous expression of their targets and the exogenous luciferase activity in constructs containing either wildtype or mutated miRNA binding sites after miRNA mimic/inhibitor transfections. A majority of miRNAs target only one anti-apoptotic family member while miR-125b, miR-133a/b, and miR-153 have been shown to target multiple members (Table 3). This implies that miR-125b, miR-133a/b, and miR-153 may have an additive effect on apoptosis compared with miRNAs that can only target one anti-apoptotic BCL2 family member. The positions of each of the discussed miRNAs are mapped on the 3′ UTR of each gene in Figure 3. In this section, we will review the miRNA-mediated post-transcriptional control of BCL2, MCL1, BCLxL, BCLW, and BFL1, and their identified roles in cancer biology and anti-cancer drug response.

### 3.1. BCL2

Thus far 12 miRNAs have been validated to target BCL2. In 2002, the miR-15/16 cluster was identified as one of the first examples of tumor suppressor miRNAs [134]. It was deleted by 13q14 deletions or downregulated by other mechanisms in approximately 70% of chronic lymphoid leukemia (CLL) patients [134]. In 2005, further studies identified that the miR-15/16 cluster directly targets *BCL2* and is now recognized as one of the major negative regulators of BCL2 expression [96]. Subsequent studies have identified that miR-155 and miR-125b also repress BCL2 through the interactions with its 3′ UTR and partially mediate proliferation and cell cycle arrest response to CD40 ligand (CD154) in human leukemia B-cells [100]. The direct repression of BCL2 by miR-125b has also been demonstrated in hepatocellular carcinoma (HCC), in which it suppresses HCC cells proliferation and induces apoptosis [99]. Recently, miR-34a has been shown to directly target the *BCL2* 3′ UTR with an inverse correlation between miR-34a and BCL2 expression levels identified in HCC tissues [98]. Functional assays have demonstrated that miR-34a inhibits cell viability, induces apoptosis, and sensitizes HCC cells to sorafenib treatment by suppressing BCL2 expression [98]. In neuroblastoma cells, miR-204 has been reported to significantly increase sensitivity to cisplatin and its expression is predictive with better clinical outcome for primary neuroblastoma tumors [102]. This effect is in part due to miR-204’s direct targeting to the 3′ UTR of *BCL2* [102]. Additionally, miR-206 has been shown to directly target BCL2 in glioblastoma cell lines and the decreased miR-206 expression is inversely correlated with the increased BCL2 expression levels in glioblastoma tissues [104]. miR-153, a brain-specific miRNA, has been reported to directly down-regulate both BCL2 and MCL1 to induce apoptosis in glioblastoma [101]. *BCL2* is also a direct target of miR-1290 and miR-206 in non-small cell lung cancer (NSCLC) [103,106], with miR-1290 mediated silencing of BCL2 has been reported to regulate the apoptotic effect to asiatic acid—a putative anti-cancer agent—in A549 cells [106]. miR-206 expression is very low in NSCLC and functions as a tumor suppressor through its downregulation of BCL2 and MET (hepatocyte growth factor receptor) [103]. In breast cancers, miR-195, miR-24-2, and miR-365-2 have been validated as negative regulators of BCL2 via their direct binding at its 3′ UTR [97]. It has been demonstrated that these miRNAs not only promote apoptosis but also enhance the apoptotic response to the anti-tumor drug etoposide in MCF7 cells [97]. Lastly, in breast cancer specimens, miR-497 levels have been shown to be inversely correlated with BCL2 expression [105]. Molecular studies have confirmed that miR-497 directly targets BCL2 and regulates apoptosis in breast cancer [105].

### 3.2. MCL1

In addition to miR-153 discussed above [101], twelve additional miRNAs have been validated to target MCL1. The first miRNA targeting MCL1, miR-29, was identified in 2007 and shown to directly and negatively regulate MCL1 expression through its 3′ UTR and control apoptosis in non-malignant H69 cholangiocytes and malignant KMCH cholangiocarcinoma cell lines [108]. Later studies have shown that miR-29 suppresses MCL1 expression and induces apoptosis in HCC [107] and ALK (anaplastic lymphoma kinase)-positive anaplastic large cell lymphoma [109]. miR-101 is another well-studied miRNA targeting MCL1. It was firstly identified that HCC commonly express low levels of miR-101 with reintroduction of miR-101 reducing MCL1 expression, thereby inducing apoptosis and suppressing tumorigenesis [114]. Further studies have reported the miR-101 induces MCL1 silencing in gastric cancer [112], NSCLC [135], endometrial cancer [113], and triple negative breast cancer (TNBC) [111]. In these studies, miR-101 not only induces apoptosis, but it is also implicated in clinical outcomes in NSCLC [135] and regulates sensitivity to paclitaxel in TNBC [111]. In addition to miR-29 and miR-101, several other miRNAs have been validated to target MCL1 in multiple cancer types in the past decade. In mouse and human hematopoietic cells, radiation induces miR-30 expression, which inhibits MCL1, but not BCL2 expression, and promotes radiation-induced apoptosis [110]. In chronic myelogenous leukemia (CML), overexpression of tyrosine-protein kinase, Lyn, has been shown to reduce miR-181 expression, which directly targets MCL1 [118]. This mechanism plays a key role for MCL1-mediated imatinib resistance in CML [118]. Likewise, ionizing radiation has been shown to induce miR-193a-3p expression and promote apoptosis by directly targeting MCL1 in U251 glioma cells [119]. In HCC, miR-125b has been shown to induce apoptosis by downregulating MCL1, BCLW, and IL6R [115]. However a microRNA screen in HCT116 colon cancer cells shows that miR-125b has no effects on MCL1 expression or on cell viability and apoptosis when treated with the BCL2 inhibitor ABT-263 [136]. These conflicting studies suggest that miR-125b-mediated MCL1 repression is cell-type-dependent and highlight the importance that specific cell-type miRNA profiles have in regulating apoptosis. In lung cancer, miR-133b expression is largely reduced compared to adjacent normal tissues and studies have shown that miR-133b targets the 3′ UTR of both *MCL1* and *BCLW* and promotes gemcitabine-induced apoptosis [117]. Also, miR-133a has been shown to target and downregulate MCL1 in osteosarcoma [116]. Additionally, miR-302b, miR-320, and miR-512-5p have been validated to target MCL1 and regulate cancer progression and apoptosis in malignant pleural mesothelioma [120], cervical cancer [121], and gastric cancer [122], respectively.

### 3.3. BCLxL

There are six miRNAs validated to target BCLxL. Let-7c/g, members of the first known human miRNA family [137,138], have been shown to negatively regulate BCLxL expression via its 3′ UTR and promote sorafenib-induced apoptosis in HCC [123]. Later reports have shown that miR-491 induces apoptosis by targeting BCLxL in colorectal cancer [139], glioblastoma [140], and ovarian cancer [126]. Further studies have reported that miR-34a and miR-608 modulate chordoma malignancy, a rare malignant tumor originating from notochord, by directly targeting BCLxL, EGFR (epidermal growth factor receptor), and MET [127]. In osteosarcoma, miR-133a not only targets MCL1 but also BCLxL where its expression level is commonly down-regulated [116]. This significantly correlates with tumor progression and patient prognosis [116]. Development of BCL2 inhibitors has revealed multiple miRNAs that regulate BCLxL expression. CLL cells resistant to ABT-199 were found to express low level of miR-377 and high level of BCLxL [125]. Further they show that miR-377 represses BCLxL expression via direct association at two binding sites in the *BCLxL* 3′UTR [125]. These findings suggest that the miR-377-mediated BCLxL repression inversely drives resistance to ABT-199 in CLL [125]. miRNA-dependent BCLxL regulation is not exclusively employed by cancers, but is also involved in normal cell homeostasis. For example, the miR-302/miR-367 cluster controls human embryonic stem cell (hESC) apoptosis by upregulating pro-apoptotic BNIP3L/Nix (BCL2/adenovirus E1B 19kDa protein-interacting protein 3-like) and downregulating BCLxL [141]. Moreover, miR-326 has been shown to target the *BCLxL* 3′ UTR and regulate apoptosis in human platelets [124].

### 3.4. BCLW

Seven miRNAs have been validated to target BCLW. miR-133b was the first validated miRNA to target BCLW in lung cancer [117]. Subsequent studies have identified that miR-203 also downregulates BCLW by directly targeting its 3′ UTR, which regulates the apoptosis response following treatment with the DNA damage agent camptothecin (CPT) in HCT116 colon cancer cells and chemosensitivity to cisplatin in bladder cancer [131,132,142]. Recently, let-7a-3p, miR-107, miR-122, miR-125b, and miR-335 have been shown to target BCLW in glioblastoma [128], NSCLC [129], pterygium [130], HCC [115], and clear cell renal cell carcinoma [133], respectively. Interestingly, although miR-184 cannot target BCLW and BCLxL in its native state, oxidation of miR-184 by reactive oxygen species (ROS) induces its association with the 3′ UTR of both *BCLW* and *BCLxL* and promotes apoptosis in heart tissue following ischemia/reperfusion injury [143]. However this study was performed in mouse models [143]. Since the 3′ UTR sequences of *BCLW* and *BCLxL* in mouse are different from those in humans, we do not know if this mechanism is maintained in a human genetic background.

### 3.5. BFL1

BFL1 has been much less well studied among the anti-apoptotic BCL2 subfamily, especially for its post-transcriptional regulation. The detailed regulation of BFL1 is reviewed in [144], but thus far no validated miRNA targeting BFL1 have been reported. A single human apoptosis array study in medullablastoma included BFL1 and identified that miR-10b downregulates BFL1 expression by approximately 31-fold [145]. BFL1 is also a predicted target of miR-181a using different bioinformatics platforms [146]. However, there have been no further studies confirming that these two miRNAs directly target BFL1. Given the importance that BFL1 may have in regulation of human BCL2 family signaling, more studies need to be carried out to build up the post-transcriptional regulation of BFL1.

## 4. Conclusions and Future Perspectives

Because of the essential roles of the BCL2 family in apoptosis regulation and cancer cell survival [19], in the past decade BH3 mimics targeting the anti-apoptotic BCL2 members have been developed from bench to bedside and back again. In 2016, ABT-199 (Venclexta), a selective BCL2 inhibitor, was approved by the U.S. Food and Drug Administration (FDA) to treat CLL in patients carrying a 17p deletion demonstrating an overall response rate of 80 percent [147]. In the meantime, several other drugs targeting single or multiple anti-apoptotic BCL2 family members have shown promising responses in single-agent or combination therapies for solid and hematopoietic tumors in pre-clinical studies and phase I/II/III clinical trials [18,148]. However, the regulatory network of the anti-apoptotic BCL2 members is still complex and unclear. In this review, we have summarized the current literature about the RBP and miRNA mediated post-transcriptional regulation of the anti-apoptotic BCL2 family. While numerous regulatory RBPs and miRNAs have been identified, the crosstalk among them has been largely ignored. A lone example is the finding that the RBP, TRA2β, competes with miR-204 binding to *BCL2* 3′ UTR to modulate *BCL2* mRNA stability [82]. Further research needs to be done to investigate the potential competitive or cooperative roles between different RBPs, and between different RBPs and miRNAs.

While the initial identification of the anti-apoptotic nature of BCL2 arose due to a chromosomal translocation resulting in its upregulation, similar genetic events have not been identified to regulate other BCL2 family members. Yet, upregulation of MCL1, BCL2, BCLxL, BCLW, and BFL1 have all been identified as drivers of tumorigenesis and chemoresistance. By understanding the post-transcriptional regulation of the anti-apoptotic family, the field will gain critical insight into the complex mechanisms that govern cellular control of apoptosis.

## Figures and Tables

**Table 1:** RNA binding protein (RBP)-mediated *BCLx* and *MCL1* alternative splicing.

Pre-mRNA	Splicing Variants	Spliceosome Components	hnRNP Proteins	SR Proteins	STAR Proteins	RBM Proteins
*BCLx*	Pro-*BCLxL* Anti-apoptotic	n.a.	hnRNP A1 [35], hnRNP A2/B1 [36]	SRSF1 [37], SRp30c [38]	n.a.	n.a.
	Pro-*BCLxS* Pro-apoptotic	SF3B1 [34]	hnRNP F [39], hnRNP H [39], hnRNP K [40], hnRNP I [41]	SRSF10 [42], SC35 [43]	SAM68 [44]	RBM4 [45], RBM10 [46], RBM11 [47], RBM25 [48]
*MCL1*	Pro-*MCL1* Anti-apoptotic	n.a.	n.a.	SRSF1 [49], SRSF2 [50]	n.a.	n.a.
	Pro-*MCL1S* Pro-apoptotic	SF3B1 [34,51], UBL5 [51], PRPF8 [51], SART [51]	n.a.	n.a.	n.a.	n.a.

n.a.: not available.

**Table 3:** Validated miRNAs targeting *BCL2*, *MCL1*, *BCLxL*, and *BCLW*.

	*BCL2*	*MCL1*	*BCLxL*	*BCLW*
Validated miRNAs	miR-15/16 [96] miR-24 [97] miR-34a [98] miR-125b* [99,100] miR-153* [101] miR-155 [100] miR-195 [97] miR-204 [82,102] miR-206 [103,104] miR-365 [97] miR-497 [105] miR-1290 [106]	miR-29 [107,108,109] miR-30 [110] miR-101 [111,112,113,114] miR-125b* [115] miR-133a* [116] miR-133b* [117] miR-153* [101] miR-181 [118] miR-193a [119] miR-302b [120] miR-320 [121] miR-512 [122]	Let-7c/g [123] miR-133a* [116] miR-326 [124] miR-377 [125] miR-491 [126] miR-608 [127]	Let-7a-3p [128] miR-107 [129] miR-122 [130] miR-125b* [115] miR-133b* [117] miR-203 [131,132] miR-335 [133]

* miRNAs targeting multiple anti-apoptotic *BCL2* family members.

## Abbreviations

AGO	Argonaute
ALK	Anaplastic lymphoma kinase
ARE	AU rich element
ASD-2	Alternative splicing defective protein 2
AUF1	AU-rich element RNA-binding protein 1
BAD	BCL2-associated death promoter
BAK	BCL2 homologous antagonist/killer
BAX	BCL2-associated X
BCLW	BCL2-like protein 2
BCLxL	B-cell lymphoma extra large
BCL2	B cell lymphoma 2
BFL1	BCL2-related protein A1
BH	BCL2 homology
BID	BH3 interacting-domain death agonist
BIM	BCL2-like protein 11
BNIP3L	BCL2/adenovirus E1B 19kDa protein-interacting protein 3-like
BOK	BCL2-related ovarian killer
CLL	Chronic lymphoid leukemia
CPT	Camptothecin
CUGBP2	CUG triplet repeat RNA-binding protein 2
EGFR	Epidermal growth factor receptor
FBI-1	Factor binding IST protein 1
GEMIN4	Gem-associated protein 4
GRE	GU rich element
H3	Histone 3
H3K4	Histone 3 lysine 4
H4	Histone 4
HCC	Hepatocellular carcinoma
HDAC	Histone deacetylase
HIF	Hypoxia inducible factor
hnRNP	Heterogeneous nuclear ribonucleoprotein
HuR	Human antigen R
KAT	Lysine acetyltransferase
LARP1	La-related protein 1
lncRNA	Long non-coding RNA
LPS	Lipopolysaccharide
LSM	Like-spliceosomal
MCL1	Myeloid cell leukemia 1
MET	Hepatocyte growth factor receptor
miRISC	microRNA induced silencing complex
miRNA	microRNA
MOMP	Mitochondrial outer membrane permeabilization
NOXA	Phorbol-12-myristate-13-acetate-induced protein
NSCLC	Non-small cell lung cancer
PEST domain	Proline, glutamic acid, serine, threonine rich domain
PNN	Pinin
Pre-mRNA	Precursor messenger RNA
PRMT2	Protein-arginine methyltransferase 2
PRPF8	Pre-mRNA-processing-splicing factor 8
PTBP1	Polypyrimidine tract binding protein 1
PUMA	p53 upregulated modulator of apoptosis
qRRM	Quasi-RNA recognition motif
RBM	RNA binding motif
RBP	RNA binding protein
RNP	Ribonucleoprotein
ROS	Reactive oxygen species
RRM	RNA recognition motif
SAM68	SRC associated in mitosis of 68 kDa
SART	Squamous cell carcinoma antigen recognized by T-cells
SF	Splicing factor
SFPQ	Splicing factor, proline and glutamine rich
siRNA	Small interfering RNA
Sm	Spliceosomal
SMN	Survival motor neuron
snRNP	Spliceosomal small nuclear ribonuclear protein
SR protein	Serine/arginine-rich protein
STAR	Signal transduction and activation of RNA
STAT	Signal transducer and activator of transcription
TNBC	Triple-negative breast cancer
TNF	Tumor necrosis factor
TRA2β	Transformer 2β
TTP	Tristetraprolin
TM domain	Transmembrane domain
UBL5	Ubiquitin-like protein 5
UTR	Untranslated region
ZFP36L1	Zinc finger protein 36, C3H1 type-like 1
